# Intermediate risk thyroid cancer management: physician survey

**DOI:** 10.1210/jendso/bvag202

**Published:** 2026-09-03

**Authors:** Annu R Suresh, Amanda Kung, Jiling Chou, Jacqueline Jonklaas

**Affiliations:** Internal Medicine Residency Program, Medstar Georgetown University Hospital, Washington, DC 20007, USA; Internal Medicine Residency Program, Medstar Georgetown University Hospital, Washington, DC 20007, USA; MedStar Center for Biostatistics, Informatics, and Data Science (CBIDS), MedStar Health Research Institute, Hyattsville, MD 20782, USA; Division of Endocrinology, Georgetown University, Washington, DC 20007, USA

**Keywords:** intermediate-risk thyroid cancer, radioactive iodine use, recurrence, pathology characteristics, physician characteristics

## Abstract

**Context:**

Variability exists in the use of radioactive iodine (RAI) treatment in intermediate-risk (IR) thyroid cancer.

**Objective:**

The study surveyed clinicians about their use of RAI in theoretical cases of patients with IR thyroid cancer.

**Design:**

A survey presented patient cases with pathology results and 2 variations consisting of baseline thyroglobulin levels of ≤5 and >5 ng/mL. Respondents were queried as to their use of RAI therapy in each case. Univariate Bayesian cumulative ordinal regression models were used to evaluate the relationships between selected predictors and the response.

**Setting:**

Online survey.

**Participants:**

American Thyroid Association members.

**Intervention:**

Participants responded regarding their management of 15 patient cases of IR thyroid cancer.

**Main outcome measures:**

Use of RAI therapy, consisting of no RAI or doses of ∼30, 40 to 74, 75 to 100, or >100 mCi.

**Results:**

Members were surveyed in May and June, 2024. Using matched vignettes, respondents were more likely to recommend RAI doses of ≥30 mCi and more likely to recommend higher RAI doses in general when thyroglobulin levels were >5 ng/mL compared with ≤5 ng/mL (odds ratios [ORs] 2.89-18.28). Examining unadjusted associations within the case scenarios presented, the presence of lymph nodes measuring 0.2-3 cm, more than 5 positive lymph nodes, and lateral lymph nodes were associated with higher RAI dose recommendations (unadjusted ORs 2.33-26.16).

**Conclusion:**

Within the IR category, there were specific pathology and biochemical findings associated with greater likelihood of the clinician recommending RAI therapy. These findings may be instructive in understanding how these features are perceived to add to the risk of thyroid cancer recurrence.

Patients with high-risk differentiated thyroid cancer (DTC) have extended survival and decreased recurrence rates with the use of radioactive iodine (RAI), whereas patients with low-risk DTC generally enjoy excellent survival and low recurrence rates without RAI being employed (1-4). Consequently, there is generally agreement regarding the use of RAI for patients with these 2 risk categories of disease. Survival rates for intermediate-risk DTC (IRDTC) are excellent (85-93%) (5), yet the relative benefits of RAI in terms of improved survival and decreased recurrence are less certain (2-4, 6-8). Furthermore, there is not full agreement as to the definition of IRDTC (4, 9). Features generally include aggressive histology, minor extrathyroidal extension, vascular invasion, and >5 lymph nodes that do not exceed 0.2 to 3 cm in size (2). Intermediate-risk DTC can also be divided into low and high intermediate risk (4, 9). Benefits from RAI treatment may only apply to a subset of intermediate-risk patients such as those who are older or who have larger tumors, or they may be more universal among this group.

Patients with DTC and their physicians face a progression of treatment decisions. Once a thyroid nodule biopsy shows cancer or high suspicion for cancer, the first decision pertains to surgery (yes/no and if yes, type: lobectomy or total thyroidectomy). Following surgery, based on the thyroid pathology, an AJCC stage (predicts survival) (10) and an American Thyroid Association (ATA) risk category (predicts recurrence risk) (11) can be assigned. Among patients who undergo a thyroidectomy, the next decision concerns RAI treatment. As noted, RAI treatment is recommended and used routinely for patients with high-risk DTC (2-4). Radioactive iodine is not currently recommended for patients with low-risk DTC and its use is decreasing (12-15). Medical teams managing individuals with low-risk DTC and high-risk DTC may thus recommend, respectively, to forgo, and have, RAI. Decisions about RAI are more complicated for people with IRDTC due to the wide spectrum of disease severity, variable risk/benefit data, and inter-physician and inter-institution differences in RAI recommendations and communication. Patient age, fertility considerations, and comorbidities also influence RAI decisions (9, 16, 17). Of note, clinical practice guidelines from the ATA published in 2016 recommend consideration of RAI for patients with IRDTC (2), an opinion also rendered in the most recent ATA guidelines (4). Within the spectrum of IRDTC, there is a trend for RAI to be utilized more often with greater disease severity (eg, greater number of involved lymph nodes, higher serum thyroglobulin levels) (18, 19). However, RAI use may also be predicted by seemingly unrelated factors such as physician perceptions and specialty, patient distance from the treating facility, and whether treatment was discussed at a multidisciplinary conference (13, 20).

This study involved administration of an online survey to members of the ATA in which they were asked whether they would recommend RAI treatment for theoretical patients with IRDTC, and if yes, what treatment dose they would select. The survey was administered prior to the publication of the 2025 ATA guidelines (4) for the management of thyroid cancer.

## Materials and methods

### Survey administration

We conducted a cross-sectional survey of the ∼1700 members of the ATA. The survey was administered using Qualtrics and was approved by the ATA survey evaluation task force. The survey was considered exempt by the Georgetown University Institution Review Board (IRB exemption ID 00007380) as it did not collect identifying data from respondents. The survey was distributed on 3 occasions by the ATA to its entire membership using distribution within a general membership email on May 14, 2024, and then using 2 stand-alone emails on May 21, 2024 and June 18, 2024. The survey was introduced with a brief statement that the investigators included trainees and that they were interested in exploring how ATA members would approach theoretical cases of patients with IRDTC with respect to their recommendations for use of RAI. The electronic survey consisted of 15 hypothetical patient scenarios, each described with relevant pathology findings, which fell within the intermediate-risk category. Due to the limited number of patient scenarios, variables other than thyroglobulin could not be independently varied across matched vignettes.

To assess the influence of thyroglobulin levels, each case was presented twice, so that matched cases could be utilized. Initially the presentation was with a baseline nonstimulated thyroglobulin level of ≤5 ng/mL, and it was then repeated with a thyroglobulin level of >5 ng/mL. This, thus, resulted in 30 paired unique clinical vignettes. For each scenario, respondents were asked to indicate their preferred RAI treatment strategy, choosing from the following options: no RAI therapy, or administration of ∼30, 40 to 74, 75 to 100, or >100 mCi of RAI. “Other” was also included as a potential response in the survey but was ultimately excluded from the analysis. Respondents also provided demographic and professional information, including medical specialty, geographic area, setting of practice, and years of experience. The survey took ∼15 to 20 minutes to complete and is shown as Table 1.

**Table 1:** Survey about intermediate-risk thyroid cancer patients administered to American Thyroid Association members

Demographic questions
1. Do you treat patients with thyroid cancer?
a. Yes (if yes, please continue the survey)
b. No (if no, please do not continue the survey. Thank you for your time)
2. How many years have you been in practice?
a. Currently in training
b. <5 years
c. 5-10 years
d. 10-20 years
e. >20 years
3. Which best describes your specialty?
a. Endocrinologist
b. Surgeon
c. Nuclear medicine physician
d. Internist
e. Other
4. In what setting do you practice?
a. Academic institution
b. Community hospital
c. Private practice
d. Other
5. Where do you practice?
a. North America
b. South America
c. Europe
d. Asia
e. Other

Survey questions*^a^*
You are presented with 15 patients with papillary thyroid cancer. Each patient scenario provides pathology results. For each patient you are asked to pick a radioactive iodine (RAI) treatment option based on the serum thyroglobulin value.

Patient 1
51-year-old female
4.2 cm focus of papillary thyroid cancer
No vascular invasion, no lymphatic invasion
No extrathyroidal extension
Surgical margins are not involved
15 central lymph nodes are removed, 0 are positive for cancer
BRAF ^V600E^ mutation is negative

Question 1a. What would you recommend for this patient if the postoperative thyroglobulin was less than or equal to 5 ng/mL?	
A	No RAI administration
B	Empiric administration of ∼30 mCi RAI
C	Empiric administration of 40-74 mCi RAI
D	Empiric administration of 75-100 mCi RAI
E	Empiric administration of >100 mCi

Question 1b. What would you recommend for this patient if the postoperative thyroglobulin was >5 ng/mL?	
A	No RAI administration
B	Empiric administration of ∼30 mCi RAI
C	Empiric administration of 40-74 mCi RAI
D	Empiric administration of 75-100 mCi RAI
E	Empiric administration of >100 mCi

Patient 2
51-year-old female
3.2 cm focus of tall cell variant of papillary thyroid cancer
No vascular invasion, no lymphatic invasion
No extrathyroidal extension
Surgical margins are not involved
15 central lymph nodes are removed, 0 are positive for cancer
BRAF ^V600E^ mutation testing is not performed

Question 2a. What would you recommend for this patient if the postoperative thyroglobulin was ≤5 ng/mL?	
A	No RAI administration
B	Empiric administration of ∼30 mCi RAI
C	Empiric administration of 40-74 mCi RAI
D	Empiric administration of 75-100 mCi RAI
E	Empiric administration of >100 mCi

Question 2b. What would you recommend for this patient if the postoperative thyroglobulin was >5 ng/mL?	
A	No RAI administration
B	Empiric administration of ∼30 mCi RAI
C	Empiric administration of 40-74 mCi RAI
D	Empiric administration of 75-100 mCi RAI
E	Empiric administration of >100 mCi

Patient 3
51-year-old female
3.2 cm focus of papillary thyroid cancer
Vascular invasion present
No lymphatic invasion
No extrathyroidal extension
Surgical margins are not involved
15 central lymph nodes are removed, 0 are positive for cancer

Question 3a. What would you recommend for this patient if the postoperative thyroglobulin was ≤5 ng/mL?	
A	No RAI administration
B	Empiric administration of ∼30 mCi RAI
C	Empiric administration of 40-74 mCi RAI
D	Empiric administration of 75-100 mCi RAI
E	Empiric administration of >100 mCi

Question 3b. What would you recommend for this patient if the postoperative thyroglobulin was >5 ng/mL?	
A	No RAI administration
B	Empiric administration of ∼30 mCi RAI
C	Empiric administration of 40-74 mCi RAI
D	Empiric administration of 75-100 mCi RAI
E	Empiric administration of >100 mCi

Patient 4
51-year-old female
4.2 cm focus of papillary thyroid cancer
No vascular invasion, no lymphatic invasion
Microscopic invasion into perithyroidal tissues
Surgical margins are not involved
15 central lymph nodes are removed, 0 are positive for cancer
BRAF ^V600E^ mutation is negative

Question 4a. What would you recommend for this patient if the postoperative thyroglobulin was ≤5 ng/mL?	
A	No RAI administration
B	Empiric administration of ∼30 mCi RAI
C	Empiric administration of 40-74 mCi RAI
D	Empiric administration of 75-100 mCi RAI
E	Empiric administration of >100 mCi

Question 4b. What would you recommend for this patient if the postoperative thyroglobulin was >5 ng/mL?	
A	No RAI administration
B	Empiric administration of ∼30 mCi RAI
C	Empiric administration of 40-74 mCi RAI
D	Empiric administration of 75-100 mCi RAI
E	Empiric administration of >100 mCi

Patient 5
51-year-old female
3.2 cm focus of papillary thyroid cancer
No vascular invasion, no lymphatic invasion
No extrathyroidal extension
Surgical margins are not involved
15 central lymph nodes are removed, 8 are positive for cancer
Largest involved lymph node is 2.7 cm in size

Question 5a. What would you recommend for this patient if the postoperative thyroglobulin was ≤5 ng/mL?	
A	No RAI administration
B	Empiric administration of ∼30 mCi RAI
C	Empiric administration of 40-74 mCi RAI
D	Empiric administration of 75-100 mCi RAI
E	Empiric administration of >100 mCi

Question 5b. What would you recommend for this patient if the postoperative thyroglobulin was >5 ng/mL?	
A	No RAI administration
B	Empiric administration of ∼30 mCi RAI
C	Empiric administration of 40-74 mCi RAI
D	Empiric administration of 75-100 mCi RAI
E	Empiric administration of >100 mCi

Patient 6
51-year-old male
3.2 cm focus of tall cell variant of papillary thyroid cancer
No vascular invasion, no lymphatic invasion
No extrathyroidal extension
Surgical margins are not involved
15 central lymph nodes are removed, 0 are positive for cancer
BRAF ^V600E^ mutation testing is not performed

Question 6a. What would you recommend for this patient if the postoperative thyroglobulin was ≤5 ng/mL?	
A	No RAI administration
B	Empiric administration of ∼30 mCi RAI
C	Empiric administration of 40-74 mCi RAI
D	Empiric administration of 75-100 mCi RAI
E	Empiric administration of >100 mCi

Question 6b. What would you recommend for this patient if the postoperative thyroglobulin was >5 ng/mL?	
A	No RAI administration
B	Empiric administration of ∼30 mCi RAI
C	Empiric administration of 40-74 mCi RAI
D	Empiric administration of 75-100 mCi RAI
E	Empiric administration of >100 mCi

Patient 7
51-year-old female
3.2 cm focus of papillary thyroid cancer
No vascular invasion, no lymphatic invasion
No extrathyroidal extension
Surgical margins are not involved
15 central lymph nodes are removed, 0 are positive for cancer
BRAF^V600E^ mutation is detected

Question 7a. What would you recommend for this patient if the postoperative thyroglobulin was ≤5 ng/mL?	
A	No RAI administration
B	Empiric administration of ∼30 mCi RAI
C	Empiric administration of 40-74 mCi RAI
D	Empiric administration of 75-100 mCi RAI
E	Empiric administration of >100 mCi

Question 7b. What would you recommend for this patient if the postoperative thyroglobulin was >5 ng/mL?	
A	No RAI administration
B	Empiric administration of ∼30 mCi RAI
C	Empiric administration of 40-74 mCi RAI
D	Empiric administration of 75-100 mCi RAI
E	Empiric administration of >100 mCi

Patient 8
51-year-old female
1.0 cm focus of papillary thyroid cancer
No vascular invasion, no lymphatic invasion
No extrathyroidal extension
Surgical margins are not involved
15 central lymph nodes are removed, 0 are positive for cancer
BRAF^V600E^ and TERT mutations are detected

Question 8a. What would you recommend for this patient if the postoperative thyroglobulin was ≤5 ng/mL?	
A	No RAI administration
B	Empiric administration of ∼30 mCi RAI
C	Empiric administration of 40-74 mCi RAI
D	Empiric administration of 75-100 mCi RAI
E	Empiric administration of >100 mCi

Question 8b. What would you recommend for this patient if the postoperative thyroglobulin was >5 ng/mL?	
A	No RAI administration
B	Empiric administration of ∼30 mCi RAI
C	Empiric administration of 40-74 mCi RAI
D	Empiric administration of 75-100 mCi RAI
E	Empiric administration of >100 mCi

Patient 9
51-year-old female
3.2 cm focus of papillary thyroid cancer
No vascular invasion, no lymphatic invasion
No extrathyroidal extension
Surgical margins are not involved
15 central lymph nodes are removed, 1 is positive for cancer
20 lateral lymph nodes are removed, 8 are positive for cancer
Largest lymph node involved is 2.7 cm in size
BRAF^V600E^ mutation is detected

Question 9a. What would you recommend for this patient if the postoperative thyroglobulin was ≤5 ng/mL?	
A	No RAI administration
B	Empiric administration of ∼30 mCi RAI
C	Empiric administration of 40-74 mCi RAI
D	Empiric administration of 75-100 mCi RAI
E	Empiric administration of >100 mCi

Question 9b. What would you recommend for this patient if the postoperative thyroglobulin was >5 ng/mL?	
A	No RAI administration
B	Empiric administration of ∼30 mCi RAI
C	Empiric administration of 40-74 mCi RAI
D	Empiric administration of 75-100 mCi RAI
E	Empiric administration of >100 mCi

Patient 10
51-year-old female
1.6 cm focus of papillary thyroid cancer in right lobe
Microscopic foci in left lobe: 1.2, 0.5, and 0.4 cm
No vascular invasion, no lymphatic invasion
No extrathyroidal extension
Surgical margins are not involved
15 central lymph nodes are removed, 0 are positive for cancer
BRAF ^V600E^ mutation is negative

Question 10a. What would you recommend for this patient if the postoperative thyroglobulin was ≤5 ng/mL?	
A	No RAI administration
B	Empiric administration of ∼30 mCi RAI
C	Empiric administration of 40-74 mCi RAI
D	Empiric administration of 75-100 mCi RAI
E	Empiric administration of >100 mCi

Question 10b. What would you recommend for this patient if the postoperative thyroglobulin was >5 ng/mL?	
A	No RAI administration
B	Empiric administration of ∼30 mCi RAI
C	Empiric administration of 40-74 mCi RAI
D	Empiric administration of 75-100 mCi RAI
E	Empiric administration of >100 mCi

Patient 11
51-year-old female
3.2 cm focus of papillary thyroid cancer
No vascular invasion, no lymphatic invasion
Microscopic invasion into perithyroidal soft tissue
Surgical margins are not involved
15 central lymph nodes are removed, 0 are positive for cancer
BRAF ^V600E^ mutation is negative

Question 11a. What would you recommend for this patient if the postoperative thyroglobulin was ≤5 ng/mL?	
A	No RAI administration
B	Empiric administration of ∼30 mCi RAI
C	Empiric administration of 40-74 mCi RAI
D	Empiric administration of 75-100 mCi RAI
E	Empiric administration of >100 mCi

Question 11b. What would you recommend for this patient if the postoperative thyroglobulin was >5 ng/mL?	
A	No RAI administration
B	Empiric administration of ∼30 mCi RAI
C	Empiric administration of 40-74 mCi RAI
D	Empiric administration of 75-100 mCi RAI
E	Empiric administration of >100 mCi

Patient 12
51-year-old female
4.2 cm papillary thyroid cancer in right lobe
Microscopic foci in left lobe: 1.2, 0.5, and 0.4 cm
No vascular invasion, no lymphatic invasion
No extrathyroidal extension
Surgical margins are not involved
15 central lymph nodes are removed, 0 are positive for cancer
BRAF ^V600E^ mutation is negative

Question 12a. What would you recommend for this patient if the postoperative thyroglobulin was ≤5 ng/mL?	
A	No RAI administration
B	Empiric administration of ∼30 mCi RAI
C	Empiric administration of 40-74 mCi RAI
D	Empiric administration of 75-100 mCi RAI
E	Empiric administration of >100 mCi

Question 12b. What would you recommend for this patient if the postoperative thyroglobulin was >5 ng/mL?	
A	No RAI administration
B	Empiric administration of ∼30 mCi RAI
C	Empiric administration of 40-74 mCi RAI
D	Empiric administration of 75-100 mCi RAI
E	Empiric administration of >100 mCi

Patient 13
51-year-old male
3.2 cm focus of papillary thyroid cancer in right lobe
Microscopic foci in left lobe: 1.2, 0.5, and 0.4 cm
No vascular invasion, no lymphatic invasion
Microscopic extension into perithyroidal soft tissue
Surgical margins are not involved
15 central lymph nodes are removed, 0 are positive for cancer
BRAF ^V600E^ mutation is negative

Question 13a. What would you recommend for this patient if the postoperative thyroglobulin was ≤5 ng/mL?	
A	No RAI administration
B	Empiric administration of ∼30 mCi RAI
C	Empiric administration of 40-74 mCi RAI
D	Empiric administration of 75-100 mCi RAI
E	Empiric administration of >100 mCi

Question 13b. What would you recommend for this patient if the postoperative thyroglobulin was >5 ng/mL?	
A	No RAI administration
B	Empiric administration of ∼30 mCi RAI
C	Empiric administration of 40-74 mCi RAI
D	Empiric administration of 75-100 mCi RAI
E	Empiric administration of >100 mCi

Patient 14
51-year-old female
3.2 cm focus of papillary thyroid cancer in right lobe
Microscopic foci in left lobe: 1.2, 0.5, and 0.4 cm
No vascular invasion, no lymphatic invasion
No extrathyroidal extension
Surgical margins are not involved
15 central lymph nodes are removed, 8 are positive for tumor
Largest involved lymph node is 2.7 cm in size
BRAF ^V600E^ mutation is negative

Question 14a. What would you recommend for this patient if the postoperative thyroglobulin was ≤5 ng/mL?	
A	No RAI administration
B	Empiric administration of ∼30 mCi RAI
C	Empiric administration of 40-74 mCi RAI
D	Empiric administration of 75-100 mCi RAI
E	Empiric administration of >100 mCi

Question 14b. What would you recommend for this patient if the postoperative thyroglobulin was >5 ng/mL?	
A	No RAI administration
B	Empiric administration of ∼30 mCi RAI
C	Empiric administration of 40-74 mCi RAI
D	Empiric administration of 75-100 mCi RAI
E	Empiric administration of >100 mCi

Patient 15
51-year-old male
4.2 cm PTC in right lobe
No vascular invasion, no lymphatic invasion
No extrathyroidal extension
Surgical margins are not involved
15 central lymph nodes are removed, 0 are positive for cancer
BRAF ^V600E^ mutation is negative

Question 15a. What would you recommend for this patient if the postoperative thyroglobulin was ≤5 ng/mL?	
A	No RAI administration
B	Empiric administration of ∼30 mCi RAI
C	Empiric administration of 40-74 mCi RAI
D	Empiric administration of 75-100 mCi RAI
E	Empiric administration of >100 mCi

Question 15b. What would you recommend for this patient if the postoperative thyroglobulin was >5 ng/mL?	
A	No RAI administration
B	Empiric administration of ∼30 mCi RAI
C	Empiric administration of 40-74 mCi RAI
D	Empiric administration of 75-100 mCi RAI
E	Empiric administration of >100 mCi

RAI, radioactive iodine.

^*a*^Other was also included as an option for each question

### Statistical analysis

#### Descriptive analyses

Descriptive analyses were performed to summarize the clinical characteristics of the IRDTC cases and the characteristics of the survey respondents.

#### Survey responses

Survey responses were coded as an ordinal variable ranging from 1 (no RAI) to 5 (>100 mCi RAI; see Table 2). Predictors included physician characteristics (experience, specialty, practice setting, and geographic area), clinical features of the hypothetical cases (patient sex, tumor size in centimeters, tall cell variant, multifocality, microscopic extrathyroidal extension, >5 positive lymph nodes, lateral lymph node involvement, presence of lymph nodes measuring 0.2-3 cm, and BRAF V600E or TERT promoter mutation), and thyroglobulin level (≤5 vs >5 ng/mL).

**Table 2:** Assigned numeric values for choice of RAI dose

Choice of RAI dose	
Numeric values	Response
1	No RAI administration
2	Empiric administration of ∼30 mCi RAI
3	Empiric administration of 40-74 mCi RAI
4	Empiric administration of 75-100 mCi RAI
5	Empiric administration of >100 mCi

RAI, radioactive iodine.

#### Univariate Bayesian cumulative ordinal regression models

We fitted Univariate Bayesian cumulative ordinal regression models with a logit link, using the *brms* package in R (21), to assess the association between the ordinal RAI dose recommendation outcome and each predictor individually. For each predictor, we estimated both proportional odds models—assuming a constant effect across thresholds of RAI dose—and nonproportional odds models, which allowed for threshold-specific effects. A random intercept for provider ID was included in all models to account for clustering of responses. Default weakly informative priors were used for regression coefficients and threshold parameters. Model comparison was performed using the Leave-One-Out Information Criterion (22); a difference in expected log predictive density greater than twice the standard error was used to select the preferred model type. Unadjusted odds ratios (ORs) with 95% credible intervals (CIs) were calculated for each predictor and interpreted as descriptive associations, reflecting unadjusted associations embedded within the case scenarios presented. For predictors best modeled with nonproportional odds, we reported threshold-specific ORs corresponding to the odds of recommending 30 mCi or higher, (2) 40 to 74 mCi or higher, (3) 75 to 100 mCi or higher, and (4) >100 mCi. For proportional odds models, a single cumulative OR was presented. This approach was used both for physician characteristics and patient case characteristics.

Response patterns were generated using 100% stacked bar plots of RAI treatment recommendations by selected tumor characteristics and thyroglobulin levels, as well as by physician characteristics. These plots were used to illustrate the distribution of clinical decision-making across different scenarios and subgroups.

#### Multivariate Bayesian cumulative ordinal regression models

Multivariable Bayesian cumulative ordinal regression models were attempted to estimate the adjusted effects of selected predictors on RAI treatment recommendations. Predictor selection was based on findings from univariate models, descriptive trends, multicollinearity assessment, and overall model performance. Adjusted ORs with 95% CIs were generated following the same format as in the univariate models. Ultimately these ORs were not reported in the manuscript itself, due to the limitation that the 15 cases included small numbers of some of the intermediate-risk characteristics and many of these were “bundled together” with other characteristics, leading to wide CIs and uncertainty. The selection of the response “other” was excluded from analysis because it did not provide a specific RAI recommendation and was deemed uninterpretable. Statistics analyses were conducted with R software 4.4.2 (23).

## Results

A total of 247 ATA members completed the survey; respondents thus constituted 15% of ATA membership. Two respondents were excluded from all analyses because of their selection of largely “other” responses to their RAI choices, and 87 “other” RAI-related responses from the rest of the respondents were also excluded (total 147 [2.0%]). The remaining 245 respondents, with 7263 observations, constituted the analytic cohort. Descriptive analyses summarizing the clinical characteristics of the IRDTC cases and the characteristics of the survey respondents are shown in Tables 3 and 4.

**Table 3:** Summary of intermediate-risk thyroid cancer characteristics of the 15 patient cases

Characteristics	*n* (%)
Sex	
Female	12 (80)
Male	3 (20)
Size of papillary thyroid cancer cm	
1	1 (6.7)
1.6	1 (6.7)
3.2	9 (60)
4.2	4 (27)
Tall cell variant	2 (13)
Multifocal	4 (27)
Microscopic extrathyroidal extension	3 (20)
More than 5 positive lymph nodes	5 (33)
Lateral nodes	1 (6.7)
Lymph node 0.2-3 cm	3 (20)
BRAF V600E or TERT mutation	
Negative	8 (53)
Not tested	4 (27)
Positive	3 (20)

Intermediate-risk thyroid cancer characteristics.

**Table 4:** Summary of physician characteristics of survey respondents

Physician demographics characteristics	Physician experience						*P*-value^b^
	Overall, *n* = 245*^a^*	Currently in training, *n* = 4*^a^*	<5 years, *n* = 16*^a^*	5-10 years, *n* = 39*^a^*	11-20 years, *n* = 91*^a^*	>20 years, *n* = 95*^a^*	
Specialty							.2
Endocrinologist	177 (73)	4 (100)	16 (100)	29 (74)	60 (67)	68 (72)	
Internist	1 (0.4)	0 (0)	0 (0)	0 (0)	0 (0)	1 (1.1)	
Nuclear medicine physician	8 (3.3)	0 (0)	0 (0)	1 (2.6)	6 (6.7)	1 (1.1)	
Other (clinical oncologist)	3 (1.2)	0 (0)	0 (0)	0 (0)	0 (0)	3 (3.2)	
Surgeon	55 (23)	0 (0)	0 (0)	9 (23)	24 (27)	22 (23)	
Unknown	1	0	0	0	1	0	
Practice setting							.8
Academic institution	206 (84)	4 (100)	14 (88)	31 (79)	77 (86)	80 (84)	
Community hospital	8 (3.3)	0 (0)	0 (0)	0 (0)	4 (4.4)	4 (4.2)	
Other (VA/government)	2 (0.8)	0 (0)	0 (0)	1 (2.6)	0 (0)	1 (1.1)	
Private practice	28 (11)	0 (0)	2 (13)	7 (18)	9 (10)	10 (11)	
Unknown	1	0	0	0	1	0	
Country							.060
Asia	29 (12)	0 (0)	0 (0)	3 (7.7)	15 (16)	11 (12)	
Europe	30 (12)	0 (0)	0 (0)	3 (7.7)	13 (14)	14 (15)	
North America	163 (67)	4 (100)	16 (100)	33 (85)	49 (54)	61 (64)	
Other	1 (0.4)	0 (0)	0 (0)	0 (0)	0 (0)	1 (1.1)	
South America	22 (9.0)	0 (0)	0 (0)	0 (0)	14 (15)	8 (8.4)	

^a^ *n* (%).

^b^Pearson χ^2^ test with simulated *P*-value (based on 100 000 replicates).

### Univariate analyses of physician characteristics and treatment choices

Radioactive iodine treatment recommendation categorizations for physician characteristics are shown in Table 2. The association between physician characteristics and the response in terms of RAI recommendations was assessed (see Table 5). Compared with the reference category of providers with >20-year experience, those providers with 11 to 20 years of experience tended to be more likely to recommend RAI doses of >100 mCi (OR = 2.09, 95% CI: 1.4-3.14). However, they did not appear to differ in their likelihood of recommending lower doses (ie, ≥30, ≥40-74, or ≥75-100 mCi). Compared with the reference category, providers with 5 to 10 years of experience tended to have a higher chance of suggesting a dose of 40 to 74 mCi or higher (OR = 1.74, 95% CI: 1.09-2.74), or 75 to 100 mCi or higher (OR = 1.67, 95% CI: 1.05-2.65). They also appeared to have an increased likelihood of recommending administration of >100 mCi (OR = 3.18, 95% CI: 1.93-5.26). Compared with the reference group, there were no apparent differences in decisions regarding dosing of RAI in the population of providers who were in training or had <5 years of experience.

**Table 5:** Univariate analysis of physician characteristics

Predictor	OR (95% CI)
Experience (Ref. = >20 years)^a^	
Experience 11-20 years	1.09 (0.78-1.54)
Experience 5-10 years	1.53 (0.99-2.35)
Experience <5 years/in training	0.95 (0.54-1.73)
Experience 11-20 years (1)	0.9 (0.63-1.3)
Experience 11-20 years (2)	0.96 (0.65-1.36)
Experience 11-20 years (3)	1.09 (0.75-1.56)
Experience 11-20 years (4)	**2.09** (**1.4-3.14)**
Experience 5-10 years (1)	0.9 (0.56-1.44)
Experience 5-10 years (2)	**1.74** (**1.09-2.74)**
Experience 5-10 years (3)	**1.67** (**1.05-2.65)**
Experience 5-10 years (4)	**3.18** (**1.93-5.26)**
Experience <5 years/in training (1)	0.78 (0.42-1.49)
Experience <5 years/in training (2)	1.08 (0.59-2.08)
Experience <5 years/in training (3)	0.64 (0.34-1.24)
Experience <5 years/in training (4)	1.89 (0.98-3.81)
Specialty (Ref. = endocrinologist)^a^	
Specialty nuclear medicine physician	1.2 (0.52-2.8)
Specialty surgeon	0.94 (0.65-1.33)
Specialty other	0.96 (0.3-3.27)
Specialty nuclear medicine physician (1)	**0.39** (**0.15-0.98)**
Specialty nuclear medicine physician (2)	0 (0-0)
Specialty nuclear medicine physician (3)	NA
Specialty nuclear medicine physician (4)	1.1 (0.36-3.29)
Specialty surgeon (1)	0.76 (0.52-1.09)
Specialty surgeon (2)	0.89 (0.61-1.3)
Specialty surgeon (3)	1.18 (0.8-1.71)
Specialty surgeon (4)	1.27 (0.83-1.92)
Specialty other (1)	0.84 (0.25-2.99)
Specialty other (2)	0 (0-0)
Specialty other (3)	NA
Specialty other (4)	0 (0-0)
Setting (Ref. = academic institution)	
Setting private practice	1.58 (0.99-2.5)
Setting other	0.58 (0.27-1.25)
Area (Ref. = North America)^a^	
Area Asia	0.87 (0.54-1.42)
Area Europe	0.81 (0.52-1.27)
Area other	1.41 (0.83-2.35)
Area Asia (1)	0.7 (0.43-1.12)
Area Asia (2)	1.02 (0.62-1.64)
Area Asia (3)	1.39 (0.85-2.26)
Area Asia (4)	**0.44** (**0.25-0.77)**
Area Europe (1)	1.17 (0.72-1.88)
Area Europe (2)	0.66 (0.41-1.05)
Area Europe (3)	0.72 (0.44-1.16)
Area Europe (4)	**0.23** (**0.12-0.42)**
Area other (1)	0.84 (0.49-1.44)
Area other (2)	1.13 (0.66-1.89)
Area other (3)	**1.87** (**1.08-3.13)**
Area other (4)	**2.81** (**1.59-4.84)**

Univariate Bayesian cumulative ordinal regression models result, proportional, and nonproportional odds model, assessing the unadjusted association between each predictor (physician characteristic) and the response scored from 1 to 5. Response ranged from “No RAI administration” = 1 to “>100 mCi” = 5. (Number of observations: 7263, number of levels (provider): 245). Bold text indicates significant values.

^a^The nonproportional odds model was preferred, unadjusted ORs were presented for both models, with nonproportional ORs reported as: (1) odds of recommending 30 mCi or higher, (2) odds of 40-74 mCi or higher, (3) odds of 75-100 mCi or higher, and (4) odds of >100 mCi. For predictors favoring proportional odds or showing no preference, only proportional odds ORs for recommending higher RAI were reported.

Compared with the reference group of endocrinologists, nuclear medicine physicians seemed to display a decrease in the likelihood of recommending doses of 30 mCi or greater (OR = 0.39; 95% CI: 0.15-0.98). Beyond this, the specialty of each respondent did not seem to be associated with differences in decisions regarding the dose of RAI administered. There were no shifts toward different decisions regarding the dose of RAI administered based on the setting in which the provider worked. Compared with the reference category of North America, providers who primarily worked in Asia appeared to have a decreased likelihood of recommending RAI doses greater than 100 mCi (OR = 0.44; 95% CI: 0.25-0.77) and providers who primarily worked in Europe appeared less likely to recommend doses >100 mCi (OR = 0.23; 95% CI: 0.12-0.42). However, in comparing the reference group to the group categorized as other areas (ie, not Europe or Asia), providers in other areas seemed more likely to recommend doses of 75 to 100 mCi or higher (OR = 1.87, 95% CI: 1.08-3.13) and doses >100 mCi (OR = 2.81, 95% CI: 1.59-4.84). As shown in Table 4, some physician characteristic categories included small numbers of physicians, which may explain the wide confidence intervals and uncertainty in the effect size estimates.

**Figure 1:**
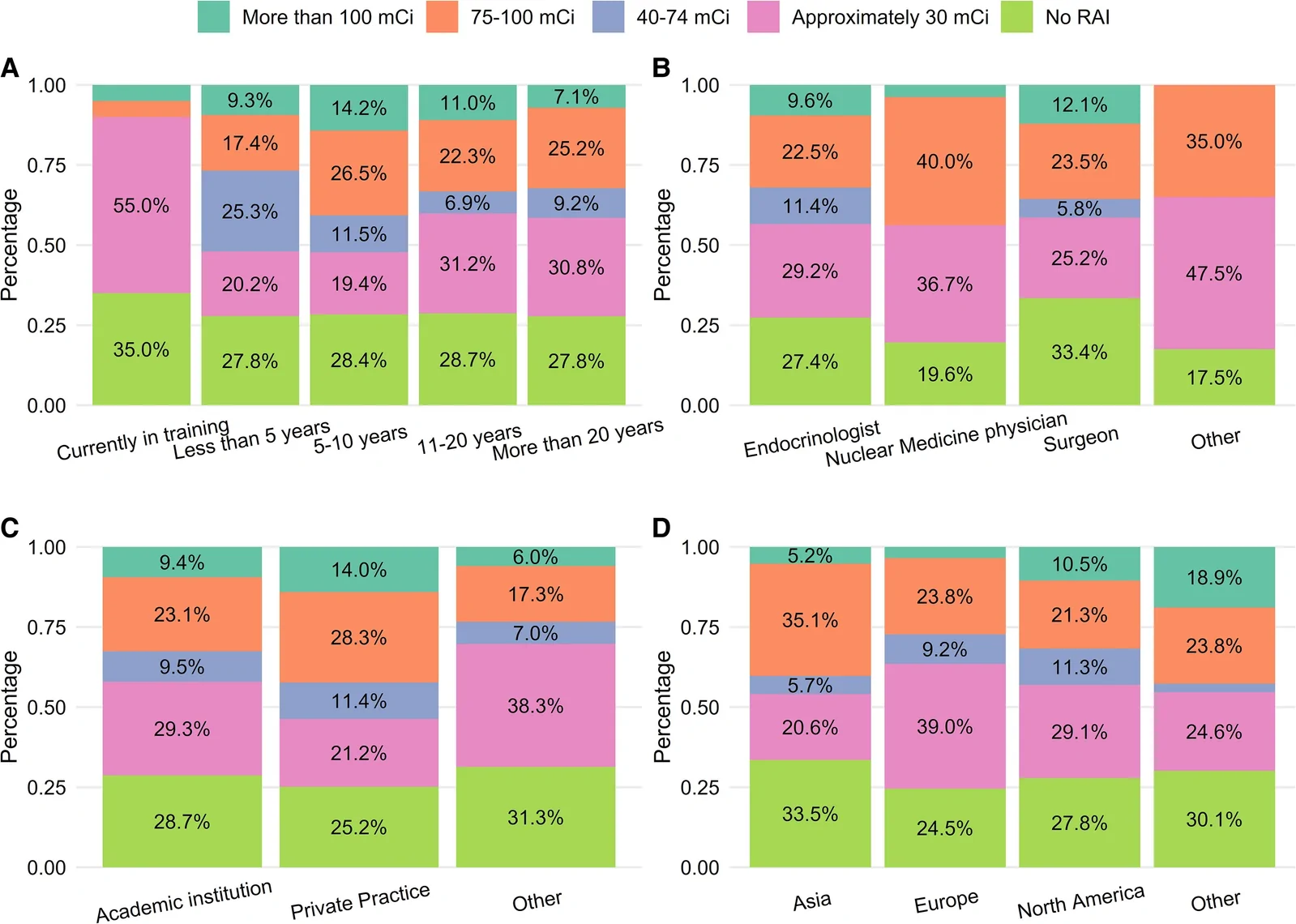
One hundred percent stacked bar plot of response in terms of RAI dose selection according to physician characteristics. Stacked bar plots show the distribution of recommended RAI dose categories according to physicians (A) experience, (B) specialty, (C) practice setting, and (D) geographic area.

Figure 1 shows the RAI treatment choices of the respondents divided by their levels of experience, specialty, practice setting, and geographic location.

### Univariate analyses of patient case characteristics and treatment choices

The association between patient characteristics (including intermediate-risk characteristics) and the response in terms of RAI recommendations was assessed (see Table 6). Throughout this analysis, the reported ORs reflect unadjusted associations, of necessity, incorporating the other characteristics within the case scenarios presented, not isolated or causal effects of individual characteristics being focused upon. Radioactive iodine treatment recommendations were categorized in an identical manner as for physician characteristics (see Table 2). Compared with female patients, male patient category was associated with recommendations to receive a lower dose of RAI overall (OR = 0.58, 95% CI: 0.52-0.64) and also associated with recommendations to receive lower doses at the level of each individual dose range. With regards to the size of the papillary thyroid cancer, there was a shift toward a decrease in suggesting 75 to 100 mCi or higher (OR = 0.92, 95% CI: 0.86-0.98) and also a shift toward a decrease in suggesting >100 mCi (OR = 0.77, 95% CI: 0.7-0.86) with an increase in tumor size of 1 cm. In addition, the presence of multifocality was associated with a decrease in the likelihood of recommending higher doses of RAI (OR = 0.79, 95% CI: 0.72-0.87). Cases with microscopic extrathyroidal extension corresponded to a shift toward a decrease in the likelihood of recommending 30 mCi or higher (OR = 0.8, 95% CI: 0.71-0.92) compared with the cases without extrathyroidal extension. There was a shift toward lower likelihood of recommending higher RAI doses for extrathyroidal extension for each of the RAI dose ranges. Specifically, there was a lower likelihood of recommending 40 to 74 mCi or higher (OR = 0.5, 95% CI: 0.43-0.57), 75 to 100 mCi or higher (OR = 0.48, 95% CI: 0.41-0.55), and 100 mCi or higher (OR = 0.31; 95% CI: 0.24-0.41).

**Table 6:** Univariate analysis of characteristics of patient case presented

Predictor	OR (95% CI)
Sex (Ref. = female)*	
Sex male	**0.58** (**0.52-0.64)**
Sex male (1)	**0.72** (**0.64-0.83)**
Sex male (2)	**0.51** (**0.45-0.59)**
Sex male (3)	**0.46** (**0.4-0.53)**
Sex male (4)	**0.42** (**0.33-0.54)**
Size of papillary thyroid cancer cm (continuous in range 1-4.2)*	
Size of papillary thyroid cancer cm	0.96 (0.92-1.01)
Size of papillary thyroid cancer cm (1)	1.04 (0.98-1.1)
Size of papillary thyroid cancer cm (2)	0.95 (0.89-1.01)
Size of papillary thyroid cancer cm (3)	**0.92** (**0.86-0.98)**
Size of papillary thyroid cancer cm (4)	**0.77** (**0.7-0.86)**
Multifocal (Ref.: no)	
Multifocal yes	**0.79** (**0.72-0.87)**
Microscopic extrathyroidal extension (Ref. = no)*	
Microscopic extrathyroidal extension yes	**0.59** (**0.53-0.65)**
Microscopic extrathyroidal extension yes (1)	**0.8** (**0.71-0.92)**
Microscopic extrathyroidal extension yes (2)	**0.5** (**0.43-0.57)**
Microscopic extrathyroidal extension yes (3)	**0.48** (**0.41-0.55)**
Microscopic extrathyroidal extension yes (4)	**0.31** (**0.24-0.41)**
More than 5 positive lymph nodes (Ref. = no)	
>5 positive lymph nodes yes	**2.33** (**2.13-2.54)**
Lateral nodes (Ref. = no)	
Lateral nodes Yes	**9.48** (**7.91-11.24)**
Lymph node 0.2-3 cm (Ref. = no)*	
Lymph node 0.2-3 cm yes	**16.34** (**14.43-18.6)**
Lymph node 0.2-3 cm yes (1)	**26.16** (**19.18-36.31)**
Lymph node 0.2-3 cm yes (2)	**20.69** (**17.43-24.49)**
Lymph node 0.2-3 cm yes (3)	**17.35** (**14.97-20.25)**
Lymph node 0.2-3 cm yes (4)	**11.24** (**9.35-13.57)**
Tall cell variant (Ref. = no)	
Tall cell variant yes	1.09 (0.96-1.23)
BRAF V600E or TERT mutation (Ref. = negative)*	
BRAF V600E or TERT mutation not tested	**2.81** (**2.54-3.12)**
BRAF V600E or TERT mutation positive	**2.23** (**1.99-2.5)**
BRAF V600E or TERT mutation not tested (1)	**2.72** (**2.36-3.15)**
BRAF V600E or TERT mutation not tested (2)	**3.02** (**2.67-3.41)**
BRAF V600E or TERT mutation not tested (3)	**2.92** (**2.55-3.32)**
BRAF V600E or TERT mutation not tested (4)	**2.95** (**2.41-3.59)**
BRAF V600E or TERT mutation positive (1)	**1.71** (**1.47-1.98)**
BRAF V600E or TERT mutation positive (2)	**2.25** (**1.96-2.59)**
BRAF V600E or TERT mutation positive (3)	**2.49** (**2.16-2.87)**
BRAF V600E or TERT mutation positive (4)	**3.38** (**2.72-4.19)**
Thyroglobulin 5 ng/mL (Ref. = less or equal to)*	
Thyroglobulin 5 ng/mL greater than	**5.65** (**5.15-6.2)**
Thyroglobulin 5 ng/mL greater than (1)	**18.28** (**15.69-21.46)**
Thyroglobulin 5 ng/mL greater than (2)	**3.58** (**3.22-3.99)**
Thyroglobulin 5 ng/mL greater than (3)	**2.89** (**2.58-3.23)**
Thyroglobulin 5 ng/mL greater than (4)	**3.39** (**2.82-4.09)**

Univariate Bayesian cumulative ordinal regression models result, proportional, and nonproportional odds model, assessing the unadjusted association between each predictor and the ordinal outcome response (RAI treatment recommendations, ranging from “No RAI administration” = 1 to “more than 100 mCi” = 5). (Number of observations: 7263, number of levels (provider): 245). Bold indicates significant values.

*The nonproportional odds model was preferred, unadjusted ORs were presented for both models, with nonproportional ORs reported as: (1) odds of recommending 30 mCi or higher, (2) odds of 40-74 mCi or higher, (3) odds of 75-100 mCi or higher, and (4) odds of >100 mCi. For predictors favoring proportional odds or showing no preference, only proportional odds ORs for recommending higher RAI were reported.

Compared with the reference of <5 positive lymph nodes, providers tended to recommend a higher dose of RAI for patients with >5 positive lymph nodes, (OR = 2.33, 95% CI: 2.13-2.54). The presence of positive lateral lymph nodes was associated with a likelihood of recommending higher doses of RAI (OR = 9.48, 95% CI: 7.91-11.24). Respondents appeared to be more likely to recommend a higher dose of RAI for patients having positive lymph nodes with a size of 0.2 to 3 cm (OR = 16.34, 95% CI: 14.43-18.6). The shift toward a recommendation for higher doses of RAI held for all dosage intervals but did not further increase for each successive dose. For cases with lymph nodes of 0.2 to 3 cm, providers tended to be more likely to recommend 30 mCi or higher (OR = 26.16, 95% CI: 19.18-36.31), 40 to 74 mCi or higher (OR = 20.69, 95% CI: 17.43-24.49), 75 to 100 mCi or higher (OR = 17.35, 95% CI: 14.97-20.25), and also 100 mCi or higher (OR = 11.24, 95% CI: 9.35-13.57).

There was no apparent difference in recommended dosing of RAI based on the presence of the tall cell variant (OR = 1.09, 95% CI: 0.96-1.23). In cases where the BRAF V600E or TERT mutations were found to be positive, providers tended toward recommendations for a higher dose of RAI (OR = 2.23, 95% CI: 1.99-2.5), compared with a negative test result. Cases where the BRAF V600E or TERT mutation were found to be positive were associated with progressively more shift toward recommending higher RAI doses. Specifically, the presence of the mutations was associated with increased tendency toward recommending at least 30 mCi (OR = 1.71, 95% CI: 1.47-1.98), ≥40 to 74 mCi (OR = 2.25, 95% CI: 1.96-2.59), ≥75 to 100 mCi (OR = 2.49, 95% CI: 2.16-2.87), and >100 mCi (OR = 3.38, 95% CI: 2.72-4.19), suggesting an upward trend in RAI dose recommendation with increasing dose thresholds. Cases in which BRAF or TERT mutation status was not tested were also associated with higher RAI dose recommendations, compared with a negative test result. However, unlike mutation-positive cases, no upward trend across dose thresholds was observed; the ORs remained ∼3.0 across all levels of RAI dosing.

Within the matched patient scenarios, the presence of a nonstimulated thyroglobulin level >5 ng/mL was associated with a 5 times increased likelihood of recommending a higher dose of RAI (OR = 5.6, 95% CI: 5.15-6.2) with providers being substantially more likely to recommend 30 mCi or higher (OR = 18.28, 95% CI: 15.69-21.46), 40 to 74 mCi or higher (OR = 3.58, 95% CI: 3.22-3.99), 75 to 100 mCi or higher (OR = 2.89, 95% CI: 2.58-3.23), and >100 mCi (OR = 3.39, 95% CI: 3.39-4.09).

**Figure 2:**
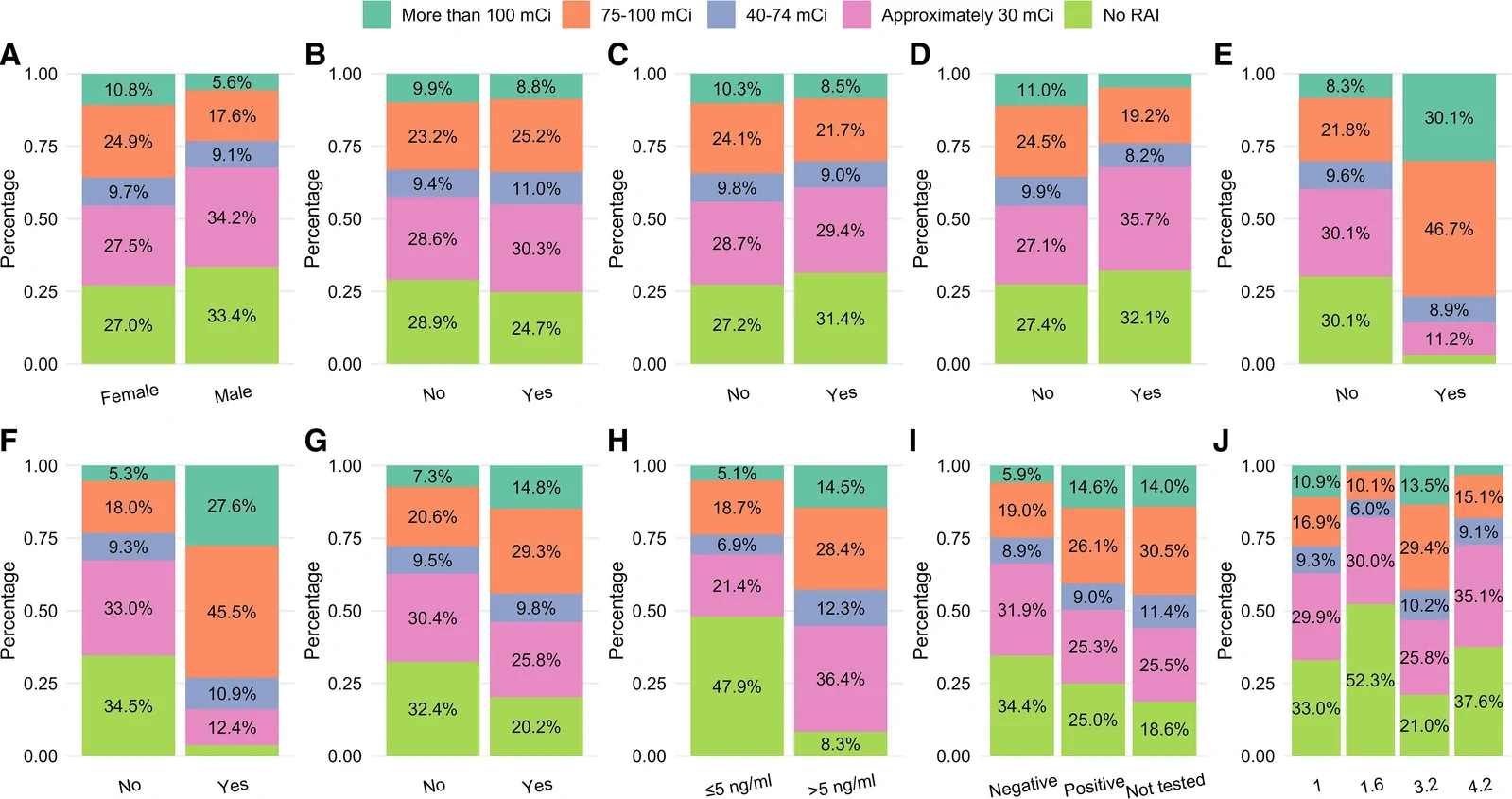
One hundred percent stacked bar plot of responses in terms of RAI dose selection according to patient sex, selected intermediate-risk thyroid cancer characteristics, and thyroglobulin concentration. Stacked bar plots show the distribution of recommended RAI dose categories according to case (A) sex, (B) tall cell variant, (C) multifocality, (D) microscopic extrathyroidal extension, (E) lateral lymph node metastasis, (F) lymph node metastasis measuring 0.2 to 3 cm, (G) ≥5 positive lymph nodes, (H) thyroglobulin level ≤5 ng/mL vs >5 ng/mL, (I) BRAF V600E/TERT mutation, and (J) papillary thyroid cancer size (cm).

Figure 2 shows the RAI doses selected by respondents displayed by patient sex and selected IRDTC characteristics and thyroglobulin concentrations.

### Multivariate analyses of physician and patient characteristics and treatment choices

Multivariate analyses examining the relationship between physician and patient characteristics, thyroglobulin levels, and RAI treatment recommendations were conducted. However, these analyses are not discussed further due to the limitations in the stability of the analysis, restricting their interpretation to possible hypothesis-gathering value.

## Discussion

The results of this survey highlight several findings with regard to clinical decision-making in the use of RAI for IRDTC.

Focusing on the unadjusted ORs from the univariate analyses and the impact of provider characteristics, it appears that clinicians with 5 to 20 years of practice may be more likely to recommend higher doses of RAI compared with those with >20 years of experience. This finding applied to both physicians with 5 to 10 and 11 to 20 years of experience. This could possibly suggest that as physicians gain more experience in IRDTC management (>20 years in practice), they tend to employ lower doses of RAI. Analyses of other physician characteristics such as specialty and geographic area, although showing some discrete findings, were suspected to be unreliable due to small numbers (nuclear medicine physicians, respondents from Asia, and “other” areas).

With regards to patient case characteristics and the findings of the unadjusted ORs from the univariate analyses, some of the findings were believed to be difficult to interpret because of small numbers (eg, patient male sex and presence of microscopic extrathyroidal extension). The same concern was true of aggressive variants and the presence of BRAF V600E or TERT mutations. Clinical scenarios including mutation positivity and mutation not tested tended to lead to recommendations for higher doses of RAI, compared with documentation of a negative mutation. The presence of affected lymph nodes and the presence of lateral lymph nodes also tended to be associated with preference for using higher doses of RAI. Some counter-intuitive findings such as lack of association with RAI dose and tumor size, and microscopic multifocality being apparently associated with recommending lower doses of RAI, were thought to be rendered unreliable by small numbers or lack of variation of these features in the 15 patient cases. These data, if they were reliable, may suggest that features such as tumor size and microscopic extra thyroidal extension are not considered sufficient indicators of recurrence risk alone or that the other characteristics discussed (thyroglobulin levels, nodal disease) may have had a stronger influence on the clinician's decision-making.

The IRDTC features that were more associated with recommendations for higher doses of RAI, using the unadjusted associations within case scenarios, included >5 positive lymph nodes, lateral nodes, and lymph nodes of 0.2 to 3 cm being present. Lymph nodes of this size appeared to be the most powerful predictor of use of higher doses of RAI. These findings are consistent with studies demonstrating that nodal burden and nodal size are predictive of recurrence and may require a more aggressive postoperative approach with use of higher dose RAI (2, 4, 16, 24-28). This approach to RAI use is also consistent with physicians generally following the guidelines prevailing at the time the surgery was conducted (2).

Although not generally included in the designation of the IRDTC category, our data suggest that clinicians place significant weight on biochemical evidence of remaining disease or completeness of surgical resection (8, 29, 30). A nonstimulated thyroglobulin level >5 ng/mL consistently led to a recommendation for RAI use across the entire range of potential doses, with the most pronounced effect being seen for recommending doses of 30 mCi RAI or higher. While the 2015 ATA guidelines for managing DTC emphasize postoperative risk stratification based on clinical and pathologic features, they also discuss completeness of resection, and our results indicate that thyroglobulin levels appear to guide decision-making, particularly in IRDTC cases where the benefit of RAI is less clearly established (2, 4, 9, 16, 17).

Limitations of this analysis include the fact that the survey was designed as vignette-paired only for the thyroglobulin levels. Other clinical characteristics were not systematically included as matched pairs. Furthermore, the large number of variables depicted in each of the 15 scenarios, likely led to the bundling of certain characteristics that may have had different impacts if fully isolated in a larger number of clinical scenarios. Unfortunately, a larger number of clinical scenarios to try to improve the survey performance would likely have lengthened the survey and led to a lower response rate. Similarly, some variables, such as the size of the lymph nodes, were only included in a small proportion of the 15 cases, leading to a small sample size for those individual variables. Only one case included vascular invasion, not allowing the effect of this characteristic to be analyzed, and the presence of a TERT mutation was only included in one case. In addition, the inclusion of “other” in the survey did not permit identification of the intended alternative decisions or allow their inclusion in the analysis. Furthermore, these cases were not separated into low-IRDTC vs high-IRDTC, a distinction that was possibly less used prior to the publication of the 2025 ATA thyroid cancer management guidelines (4). Additional limitations relate to whether the physician responses were representative of the management strategy of thyroidologists in general. Only 15% of ATA members responded to this surgery, suggesting that the results of this survey may not be fully generalizable. In addition, some of the respondent categories (eg, nuclear medicine specialty) were small.

Overall, this study explores the IRDTC characteristics that seem to drive decision-making in favor of higher doses of RAI. The most robust finding is the association of higher thyroglobulin levels being associated with choice of higher doses of RAI. A nonstimulated thyroglobulin level >5 ng/mL consistently led to a recommendation of RAI use across the entire range of doses.

Based on unadjusted associations within the case scenarios, other influences seem to be lymph node presence and lymph node number. Another feature appeared to be physician experience of <20 years which seemed to be associated with more use of higher doses of RAI therapy. However, these latter characteristics cannot be considered major predictors, as they were unadjusted associations.

## Data Availability

Some or all datasets generated during and/or analyzed during the current study are not publicly available but are available from the corresponding author on reasonable request.

## Prior presentation

The manuscript was submitted as an abstract to the 2025 Annual Meeting of the American Thyroid Association.

## Abbreviations

ATA: American Thyroid Association
CIs: credible intervals
DTC: differentiated thyroid cancer
IR: intermediate risk
OR: odds ratio
RAI: radioactive iodine
